# Environmental influences on children's physical activity

**DOI:** 10.1136/jech-2014-204287

**Published:** 2014-10-30

**Authors:** Theodora Pouliou, Francesco Sera, Lucy Griffiths, Heather Joshi, Marco Geraci, Mario Cortina-Borja, Catherine Law

**Affiliations:** 1 School of Social and Community Medicine, University of Bristol, Bristol, UK; 2 UCL Institute of Child Health, London, UK; 3 Department of Quantitative Social Science, Institute of Education University of London, London, UK; 4 Department of Epidemiology and Biostatistics, Arnold School of Public Health, University of South Carolina, Columbia, South Carolina, USA

**Keywords:** PHYSICAL ACTIVITY, SOCIO-ECONOMIC, ENVIRONMENTAL HEALTH, CHILD HEALTH, SOCIAL INEQUALITIES

## Abstract

**Background:**

This paper aims to assess whether 7-year-olds’ physical activity is associated with family and area-level measures of the physical and socioeconomic environments.

**Methods:**

We analysed the association of environments with physical activity in 6497 singleton children from the UK Millennium Cohort Study with reliable accelerometer data (≥2 days and ≥10 h/day). Activity levels were assessed as counts per minute; minutes of moderate to vigorous activity (MVPA); and whether meeting recommended guidelines (≥60 min/day MVPA).

**Results:**

Higher levels of children's physical activity were associated with households without use of a car and with having a television in a child's bedroom (for counts per minute only). Aspects of the home socioeconomic environment that were associated with more children's physical activity were lone motherhood, lower maternal socioeconomic position and education, family income below 60% national median, and not owning the home. Children's activity levels were higher when parents perceived their neighbourhood as poor for bringing up children and also when families were living in the most deprived areas. Relationships were independent of characteristics such as child's body mass index and ethnic group. When adjusted for physical and socioeconomic correlates, the factors remaining significant in all outcomes were: household car usage and maternal education.

**Conclusions:**

Although physical and socioeconomic environments are associated with children’s physical activity, much of the variation appears to be determined by the child's home socioeconomic circumstances rather than the wider environment where they live.

## Introduction

Physical activity is health enhancing, and is associated with both reduced risk of adiposity, diabetes, hypertension, musculoskeletal problems and promotion of psychological well-being.^1^ Rates of activity are low among UK children, particularly girls. Using accelerometer data, the 2008 Health Survey for England (HSE) found that 51% of boys and 34% of girls aged 4–10 years met the current minimum physical activity recommendations,^2^ although it is important to recognise that current recommendations are based on evidence on self-reported physical activity. Equivalent figures for 7-year-olds in the Millennium Cohort Study (MCS) were 63% and 38%.^3^ Understanding what influences children's physical activity may help to identify interventions to promote active lifestyles.

Observational studies relating physical activity between environmental factors give inconsistent findings. Some find an association,^4–12^ others do not.^13^ ^14^ These differences may be due to limitations in study design: with one exception,^15^ most studies have a small sample size (less than 150 children^4^ ^5^ ^10^), focus on population subgroups^5^ ^13^ or, as noted in a recent review, rely on reports of physical activity, sometimes by parents.^16^ Recent studies have also emphasised adolescents rather than children and most are based in North America or Australia.^16^

Differences between studies may also be due to the environmental measures used. Most research focuses on either perceived or objective measures of the physical environment and only two studies have examined both.^6^ ^10^ Furthermore, children experience their environments at different levels, such as the immediate home environment as well as its neighbourhood. Environments may be characterised in different ways, for example, in socioeconomic and physical terms. Most studies to date have focused on the built environment of the neighbourhood; they show that children are more likely to be physically active if their neighbourhood has facilities such as walking/cycling paths and parks^8–10^; playgrounds and recreational community centres^4^ ^9^ ^17^ and features sidewalks, lighting, street connectivity or land-use diversity.^5^ ^6^ ^8^ ^10^ ^17^ The few studies to examine the association between children's physical activity and rurality have shown mixed results.^18^ Questions also remain on the mechanisms through which the built and socioeconomic environments exert influences on physical activity as few studies have controlled for individual socioeconomic factors.^7^ ^9–12^

The present study addresses several of these research gaps. Using data on 7-year-old children from a large, nationally representative UK cohort, it explores the influence of characteristics of the home and neighbourhood environments on accelerometer-measured physical activity, taking account of family socioeconomic circumstances and using measures that reflect physical and social characteristics of the neighbourhood, objective and subjective.

## Methods

### Participants

The MCS is a UK-wide prospective study of children born between September 2000 and January 2002. The original cohort comprised of 18 818 children (72% response rate) whose parents were first interviewed when their child was aged 9 months.^19^ Three more home interviews were carried out at ages 3, 5 and 7 years with further follow-up conducted at 11 years (data not available at time of analysis) and beyond. Detailed information regarding demographic, social, and health factors relating to the children, and the children's siblings and parents was obtained through interviews of the main respondents and their partners in the home.^20^ This study uses data from the age seven survey, which received ethical approval from the Northern and Yorkshire Research Ethics Committee (07/MRE03/32). The present analysis did not require additional ethics approval.

### Physical activity data

At age 7, 14 043 children (13 681 singletons) were interviewed and invited to participate in the accelerometry study. Those who consented were asked to wear the Actigraph GT1M uniaxial accelerometers (Actigraph, Pensacola, Florida). Previous studies have demonstrated this device to be a technically reliable instrument, able to detect differing levels of physical activity intensity.^21^ Accelerometers programmed to use a 15 s sampling epoch and to record activity as counts and steps were sent to those who consented to participate (n=12 768 singletons). Children were instructed to start wearing their accelerometer the morning after receiving it and to do so for seven consecutive days during waking hours, except during bathing/aquatic activities. Data were collected between May 2008 and August 2009. Accelerometers were returned from 9980 children (9721 singletons). Data from the activity monitors were downloaded using the Actigraph software V.3.8.3 (Actigraph, Pensacola, Florida, USA) and processed in house,^22^ according to predetermined criteria.^23^ Non-wear time was defined as any time period of consecutive zero-counts ≥20 min and these periods were removed from the summation of activity. A threshold for extreme values was set to ≥11 715 counts and time spent at intensity above this threshold was excluded.^24^ Participants with recording periods of ≥10 h on ≥2 days were included in analyses,^23^ resulting in a sample of 6497 singleton children. Small differences were found in the demographic characteristics of the sample of children with reliable accelerometer data (n=6497) relative to the whole cohort sample (n=13 681 singletons) interviewed at age 7 years.^3^ To allow for possible bias in the selection of children participating in the accelerometry sample, an inverse probability weight was applied.^25^ This was in addition to the standard weighting applied to all cohort children to allow for the original sampling design and attrition.

The following outcome variables were derived: total physical activity (mean daily counts per minute (cpm) of wearing time, mean daily minutes of moderate to vigorous activity (MVPA) and adherence to current recommended guidelines (at least 60 min MVPA per day). The cut-off classifying physical activity as moderate-to-vigorous (>2241 cpm) was defined according to a calibration study in children of similar age, testing a range of activities from sedentary (eg, sitting) to vigorous (eg, basketball and jogging).^26^ These measures were standardised by introducing the notion of a standard day with equal duration (735 min, equal to mean wear time across all reliable days), minimising in this way the potential association between physical activity and wearing time.^25^

### Explanatory variables

We examined the influence of a number of environmental factors: all collected at the age 7 survey (fourth sweep of the MCS survey). Gender, season (based on the astronomical definition: spring (21 March–20 June), summer (21 June–20 September), autumn (21 September, 20th December), winter (21 December–20 March), ethnic group^27^ and body mass index (BMI) of the children (based on measured height and weight information and categorised according to the International Obesity Task Force (IOTF) cut-offs for children) were included.^28^ To minimise loss of information questionnaire items missing at age 7 years were retrieved from the previous sweep (age 5 years) if available.

#### Home environment (reported measures)

- *The physical environment* was represented by the type of accommodation (house/bungalow; flat, studio or maisonette and bedsit or other), number of household cars/vans in regular use, whether participants had access to a garden, and whether the child had a television (TV) in their bedroom.
- *The socioeconomic environment* was represented by lone motherhood (being a lone mother or not); housing tenure (own/mortgage or other); family size (only child or not). We also included socioeconomic circumstances of the mother on the National Statistics Socioeconomic Classification, grouped into four categories: managerial and professional; intermediate occupations; routine and manual occupations and never worked or long-term unemployed.^29^ Maternal education was divided into two groups: at or above O-level (or equivalent)/below O-level. Poverty was defined by whether family income was <60% of the national median, before housing costs but after benefits and using a modified Organisation for Economic Co-operation and Development (OECD) equivalence scale.^30^

#### Neighbourhood (reported and objective measures)

- *The physical environment* was represented by the following reported measures: accessibility to play areas and whether the area in which they lived (defined as one mile or 20 min walk from their house) was perceived to be good and safe to raise children. In addition, the objectively measured 2005 Rural/Urban Area Classification (RUAC) at the Lower Super Output Area level (LSOA; an average of 1500 people) was included in the analysis.^31^ The RUAC categories were: urban (>10 000); rural, which included village, hamlet and isolated dwellings.
- *The socioeconomic environment* was represented by the objectively measured 2004–2005 Index of Multiple Deprivation (IMD) at the LSOA level.^31^ The IMD measures relative levels of deprivation in small areas based on a number of indicators. The indicators are income, employment, health deprivation and disability, education, skills and training, barriers to housing and services, crime and living environment. As there is no unified definition for these measures across the UK, these are held as country-specific variables. While the IMD definitions are not directly equivalent, they could be broadly compared by introducing in addition to the main UK country and IMD variables, an interaction term between UK country and IMD. For the purposes of this study we used the IMD country-specific quintiles. This parameterisation allows us to compare the higher quintiles (more deprived) of each country with the country-specific IMD reference category.

### Statistical analysis

Analyses were performed using STATA/SE V.12.0 (Stata Corporation, Texas, USA). Sampling weights were used to account for the stratified clustered design of the MCS. Weights were adjusted for attrition between contacts at successive MCS sweeps and for missing accelerometer data. Details on the adjustment for non-response and non-compliance are given elsewhere.^25^ Total activity and MVPA were log-transformed to account for their positively skewed distributions. For each regression coefficient b, we calculated the quantity 100×(e^b^ −1); similarly, the lower and upper bounds of b*’*s 95% CI were subject to the same back-transformation. These values can be interpreted as the percentage change between geometric means of total activity or time spent in MVPA associated with varying levels of the covariates of interest. The p values were calculated using the command nlcom in Stata, which is based on the delta method to approximate nonlinear combinations of parameter estimates.^32^

Regression models examined the association between characteristics of the home and neighbourhood environments and the three outcomes describing children's physical activity. Considering the stratified cluster sampling design of MCS study, weights to adjust for attrition between contacts at successive MCS sweeps and for missing accelerometer data were taken into account during the estimation using the Stata command svyset. Linear regression models were fitted to analyse total activity (cpm) and MVPA, while logistic regression analysis was used for activity adherence. Analyses were repeated separately for each outcome using two different models: model 1 was adjusted for gender and season; model 2 was further adjusted for children's BMI and ethnic group.

Two-level linear and logistic regression models (models 1 and 2) were also fitted to examine the relationships between physical activity and objective measures of the neighbourhood environment. The two levels of analysis were family and the electoral wards (or superwards). Families were considered as the first level of analysis to account for contacts at successive MCS sweeps and for missing accelerometer data. The wards were defined as the second level of analysis in our study to account for the MCS sampling design. Aforementioned, with main effects for UK country and IMD we included an interaction term between each UK country and country-specific IMD in the multilevel models.

Two-level linear and logistic regression models (model 3) were, in addition to gender, season, children's BMI and ethnic group, adjusted for environmental characteristics and objective measures of the neighbourhood environment that were statistically significant in models 1 and 2. Multicollinearity was assessed using the variance inflation factor for each estimator (for individual and area levels of analysis). Random intercept-only multilevel models were fitted using gllamm, a Stata programme for mixed-effects modelling.^33^ The intracluster correlation coefficients (ICC) from the multilevel models were used to quantify the amount of variation in measures of physical activity resulting from differences between areas.

As a sensitivity analysis, we repeated analyses separately for boys and girls; results were not different to those presented here. Characteristics of the children and their families and reported measures of home-environments and neighbourhood-environments showed no difference between non-movers and those who moved between contacts at 5 and 7 years (563 children; results not shown). Excluding children who moved between contacts at 5 and 7 years from the analysis did not affect the associations (data not shown).

## Results

Table 1 shows the characteristics of the children in the sample, and the physical and socioeconomic characteristics of their home and neighbourhood environments. Although most children appeared to be in relatively advantaged circumstances (eg, their family had the use of a car or owned their own home), the sample was diverse: for example, 22.0% were lone mothers. Just over half of the children had a TV in their bedroom and around a fifth were overweight or obese. Most families were living in a neighbourhood with good access to play areas (90.4%), and were generally satisfied with the neighbourhood (71.8%). Descriptive statistics for all physical activity variables and sedentary time have been previously published elsewhere.^3^

**Table 1:** Descriptive characteristics of the sample based on reported measures unless otherwise stated: number (weighted %) of singletons at age 7 for all children with reliable accelerometry data

Reliable accelerometry data sample (6497 children)	
*Child's general characteristics*	
Gender	
Boys	3176 (50.9)
Girls	3321 (49.1)
Child's ethnic group	
Caucasian	5710 (85.2)
Mixed	168 (3.2)
Indian	139 (2.0)
Pakistani	177 (3.8)
Bangladeshi	70 (1.3)
African-American	142 (2.8)
Other ethnic group	90 (1.6)
Child's BMI	
Optimal	5310 (80.0)
Overweight	827 (14.0)
Obese	283 (5.9)
*Home*	
*Physical environment*	
Type of accommodation	
House or bungalow	5838 (86.4)
Flat, studio, bedsit or other	656 (13.6)
Child has a TV in his/her bedroom	3046 (52.7)
Access to garden	6179 (92.5)
Cars in use	
None	579 (14.3)
One	2304 (37.1)
More than one	3604 (48.6)
*Socioeconomic environment*	
Housing tenure	
Own/mortgage	4873 (62.5)
Other	1614 (37.5)
Number of children in the household	
Only child	726 (12.4)
At least one other child	5771 (87.6)
Lone mother	961 (22.0)
Maternal socioeconomic class	
Managerial and professional occupations	2189 (34.1)
Intermediate occupations	1201 (19.5)
Routine and manual occupations	2582 (42.5)
Never worked and long-term unemployed	268 (3.9)
Maternal education	
At or above O-level	5015 (69.0)
Below O-level	1482 (31.0)
Poverty (<60% median income)	1462 (30.2)
*Neighbourhood*	
*Physical environment*	
Access to play areas	5824 (90.4)
Good area to bring up children	
Excellent/good	4806 (71.8)
Average	1180 (21.8)
Poor/very poor	316 (6.4)
Parental perception of safety	
Very safe/fairly safe	5608 (86.4)
Neither safe nor unsafe	439 (8.3)
Fairly unsafe/very unsafe	259 (5.3)
Urban/rural (objective)	
Urban (>10k)	12 203 (86.8)
Rural	1830 (13.2)
*Socioeconomic environment (objective)*	
Index of multiple deprivation England	
Least deprived	912 (20.1)
Second	821 (19.3)
Third	830 (20.5)
Fourth	789 (18.4)
Most deprived	848 (21.8)
Index of multiple deprivation Wales	
Least deprived	243 (27.4)
Second	154 (17.8)
Third	126 (12.4)
Fourth	193 (19.5)
Most deprived	183 (23.0)
Index of multiple deprivation Scotland	
Least deprived	215 (21.1)
Second	163 (19.8)
Third	169 (22.2)
Fourth	122 (19.8)
Most deprived	92 (17.2)
Index of multiple deprivation Northern Ireland	
Least deprived	129 (18.1)
Second	117 (18.5)
Third	130 (19.0)
Fourth	140 (20.8)
Most deprived	120 (23.6)

Missing data: child's ethnic group, 1; child's BMI (based on measured height and weight and classified according to the International Obesity Task Force; IOTF), 77; type of accommodation, 3; child has TV in his/her bedroom, 7; access to gardens, 3; household cars in use, 10; housing tenure, 10; maternal education, 242; access to play areas, 12; good area to bring up children, 195; parental perception of safety, 191; urban/rural, 1 (England) and Index of Multiple Deprivation, 1 (England).

BMI, body mass index; TV, television.

### Home environmental measures

More cars in use in the household were significantly associated with less children's physical activity in unadjusted and adjusted analyses. In unadjusted analyses, children who had a TV in their bedroom were more physically active and more likely to meet activity guidelines. This association was attenuated after adjustment for all significant correlates of the home and neighbourhood but remained statistically significant for counts per minute. Type of accommodation and access to gardens were not consistently associated with physical activity (table 2).

**Table 2:** Differences in children's physical activity by reported and objective measures of the neighbourhood environment (models run on all singleton children with reliable activity data (n=6497))

	Counts per minute			Moderate to vigorous physical activity			Activity adherence (OR and 95% CI)		
	Model 1	Model 2	Model 3	Model 1	Model 2	Model 3	Model 1	Model 2	Model 3
*Home*									
Physical environment									
Type of accommodation									
House or bungalow (reference category)									
Flat, studio, bedsit or other	1.51	2.15		3.17	2.86		1.14	1.14	
	−1.32 to 4.33	−0.63 to 4.92		−0.73 to 7.06	−1.10 to 6.82		0.94 to 1.38	0.93 to 1.39	
Access to gardens	−2.77	−3.70		−5.75*	−5.40*	−4.20	0.71 *	0.72*	0.69
	−6.63 to 1.08	−7.48 to 0.09		−10.98 to −0.52	−10.68 to −0.11	−8.66 to 0.26	0.52 to 0.97	0.51 to 1.00	0.46 to 1.04
Cars in use									
None (reference category)									
One	−4.6**	−4.90***	−5.05**	−7.27***	−7.58***	−7.68***	0.60***	0.58***	0.53***
	−7.33 to −1.87	−7.57 to −2.23	−8.10 to −2.00	−10.88 to −3.66	−11.24 to −3.91	−11.93 to −3.42	0.47 to 0.77	0.45 to 0.75	0.38 to 0.73
More than 1	−6.88***	−7.65***	−7.58***	−11.29***	−11.75***	−11.8***	0.45***	0.43***	0.39***
	−9.59 to −4.17	−10.21 to −5.10	−10.96 to −4.20	−14.81 to −7.78	−15.17 to −8.34	−16.42 to −7.18	0.35 to 0.58	0.33 to 0.56	0.28 to 0.56
Child has a TV in his/her bedroom	4.13***	3.63***	2.01*	4.41**	4.35**	1.98	1.22**	1.24**	1.04
	2.39 to 5.88	1.91 to 5.35	0.37 to 3.65	1.89 to 6.94	1.81 to 6.90	−0.40 to 4.36	1.09 to 1.38	1.09 to 1.40	0.89 to 1.23
Socioeconomic environment									
Lone mother	3.88**	3.86**	−0.54	6.35***	6.11***	−0.41	1.37**	1.37**	1.03
	1.65 to 6.11	1.64 to 6.09	−2.98 to 1.89	3.14 to 9.57	2.91 to 9.31	−3.81 to 2.98	1.13 to 1.66	1.13 to 1.66	0.80 to 1.33
Family size: only child	−0.54	−0.26		−0.19	0.36		1.13	1.18	
	−2.59 to 1.52	−2.41 to 1.89		−3.23 to 2.85	−2.82 to 3.54		0.93 to 1.38	0.96 to 1.45	
Housing tenure: own/mortgage	−3.03**	−3.37***	1.18	−4.64***	−4.74***	1.59	0.77**	0.75**	1.20
	−4.74 to −1.32	−5.05 to −1.69	−0.98 to 3.25	−7.02 to −2.27	−7.14 to −2.34	−1.49 to 4.68	0.65 to 0.90	0.63 to 0.88	0.95 to 1.51
Maternal socioeconomic circumstances									
Managerial and professional occupations (reference category)									
Intermediate occupations	1.31	1.31	−0.38	1.09	0.99	−1.25	1.01	1.01	0.90
	−0.73 to 3.34	−0.73 to 3.35	−2.40 to 1.65	−1.92 to 4.10	−2.04 to 4.02	−4.26 to 1.75	0.84 to 1.20	0.84 to 1.21	0.73 to 1.09
Routine and manual occupations	2.07*	2.38**	−0.83	2.11	2.42	−2.26	1.15	1.17*	0.94
	0.25 to 3.89	0.60 to 4.17	−2.67 to 1.01	−0.55 to 4.76	−0.24 to 5.08	−5.06 to 0.54	0.98 to 1.35	1.00 to 1.37	0.78 to 1.15
Never worked and long-term unemployed	1.80	5.74**	−2.19	4.08	8.01*	−5.27	1.46*	1.75**	0.83
	−2.41 to 6.02	1.51 to 9.97	−6.32 to 1.94	−2.03 to 10.18	1.28 to 14.74	−11.89 to 1.35	1.01 to 2.12	1.19 to 2.59	0.51 to 1.35
Maternal education									
At or above O-level (reference category)									
Below O-level	1.97 *	3.22**	2.58*	2.99*	4.27**	3.40*	1.20*	1.28**	1.30*
	0.19 to 3.75	1.40 to 5.04	0.36 to 4.80	0.39 to 5.59	1.69 to 6.85	0.06 to 6.75	1.03 to 1.39	1.09 to 1.50	1.03 to 1.65
Poverty (<60% median income)	2.57*	3.74***	−0.11	4.58**	5.53***	−0.41	1.26**	1.32**	0.81
	0.55 to 4.58	1.82 to 5.66	−2.32 to 2.11	1.50 to 7.66	2.55 to 8.51	−3.75 to 2.93	1.06 to 1.49	1.11 to 1.57	0.62 to 1.06
*Neighbourhood*									
Physical environment									
Access to play areas	0.80	0.96		0.40	0.88		0.93	0.97	
	−1.68 to 3.29	−1.54 to 3.47		−3.24 to 4.03	−2.77 to 4.54		0.73 to 1.18	0.76 to 1.24	
Good area to bring up children									
Excellent/good (reference category)									
Average	1.41	1.67	−0.30	3.06*	3.13*	0.01	1.13	1.14	0.96
	−0.66 to 3.49	−0.29 to 3.64	−2.21 to 1.60	0.13 to 6.00	0.28 to 5.99	−2.84 to 2.87	0.96 to 1.33	0.97 to 1.35	0.80 to 1.15
Poor/very poor	3.63*	4.04*	0.69	7.32*	7.17*	2.01	1.25	1.24	0.96
	0.00 to 7.28	0.36 to 7.72	−3.26 to 4.64	1.35 to 13.29	1.27 to 13.07	−4.01 to 8.04	0.93 to 1.69	0.92 to 1.69	0.62 to 1.50
Parental perception of safety									
Very safe/fairly safe (reference category)									
Neither safe nor unsafe	0.54	0.73		2.09	2.06		1.17	1.16	
	−2.21 to 3.29	−2.00 to 3.47		−2.26 to 6.43	−2.28 to 6.39		0.91 to 1.49	0.90 to 1.49	
Fairly unsafe/very unsafe	1.34	1.33		2.05	1.46		1.05	1.05	
	−2.42 to 5.10	−2.41 to 5.07		−4.14 to 8.24	−4.52 to 7.44		0.77 to 1.45	0.77 to 1.44	
Urban/rural morphology (objective)									
Urban (>10k; reference category)									
Rural	−0.81	−0.93	0.93	−2.31	−2.13	−0.01	0.85	0.87	1.01
	−2.70 to 1.08	−2.82 to 0.95	−1.00 to 2.87	−5.12 to 0.51	−4.96 to 0.70	−2.94 to 2.92	0.69 to 1.04	0.71 to 1.07	0.82 to 1.24
*Intraclass correlation (ICC)(%)*	4.49	4.44	4.06	4.06	4.09	3.62	5.03	5.60	5.45
Socioeconomic environment									
Index of Multiple Deprivation (objective)									
England									
Least deprived (reference category)									
Second	1.82	1.78	0.97	2.16	2.05	1.16	1.11	1.10	1.02
	−0.62 to 4.26	−0.65 to 4.22	−1.49 to 3.43	−1.61 to 5.92	−1.75 to 5.84	−2.66 to 4.98	0.87 to 1.42	0.86 to 1.41	0.79 to 1.32
Third	0.92	1.13	0.06	1.21	1.47	−0.09	1.09	1.12	1.02
	−1.51 to 3.35	−1.26 to 3.51	−2.34 to 2.46	−2.41 to 4.84	−2.15 to 5.08	−3.71 to 3.53	0.86 to 1.39	0.88 to 1.43	0.79 to 1.31
Fourth	3.82*	4.26**	1.33	6.43**	6.94**	2.65	1.39**	1.43**	1.12
	0.88 to 6.75	1.36 to 7.17	−1.81 to 4.47	2.06 to 10.79	2.57 to 11.31	−2.01 to 7.32	1.09 to 1.76	1.11 to 1.84	0.83 to 1.51
Most deprived	4.97**	6.16***	2.65	7.95**	8.94***	3.42	1.67**	1.78***	1.34
	1.23 to 8.71	2.82 to 9.51	−0.90 to 6.19	2.72 to 13.19	3.95 to 13.93	−1.70 to 8.53	1.23 to 2.27	1.31 to 2.43	0.94 to 1.92
Wales									
Least deprived (reference category)									
Second	−1.68	−1.79	−0.37	0.39	0.23	2.97	0.77	0.76	0.80
	−6.84 to 3.48	−6.78 to 3.20	−5.60 to 4.87	−7.12 to 7.91	−6.86 to 7.31	−5.37 to 11.31	0.26 to 1.28	0.32 to 1.21	0.31 to 1.29
Third	−0.61	−0.40	−0.01	−2.78	−2.22	−1.26	0.63*	0.66	0.69
	−7.60 to 6.38	−7.45 to 6.66	−7.61 to 7.59	−13.04 to 7.49	−12.68 to 8.24	−12.55 to 10.03	0.28 to 0.98	0.27 to 1.04	0.27 to 1.10
Fourth	−1.07	−0.68	−0.70	0.80	1.34	1.72	0.99	1.03	1.04
	−6.47 to 4.32	−6.45 to 5.10	−7.03 to 5.62	−7.40 to 9.01	−7.70 to 10.37	−8.23 to 11.67	0.46 to 1.53	0.46 to 1.60	0.41 to 1.67
Most deprived	0.44	0.46	−0.88	2.00	2.02	0.80	0.84	0.84	0.79
	−4.62 to 5.51	−4.65 to 5.56	−6.18 to 4.43	−5.16 to 9.16	−5.29 to 9.32	−6.75 to 8.36	0.27 to 1.41	0.26 to 1.41	0.23 to 1.34
Scotland									
Least deprived (reference category)									
Second	2.99	2.66	1.79	4.51	4.68	3.82	1.11	1.13	1.15
	−1.97 to 7.94	−2.47 to 7.78	−3.47 to 7.04	−3.23 to 12.26	−3.36 to 12.71	−4.47 to 12.11	0.53 to 1.69	0.50 to 1.76	0.52 to 1.77
Third	−1.17	−0.30	−0.84	−1.78	−0.31	−1.01	0.72	0.83	0.82
	−6.78 to 4.45	−6.04 to 5.44	−6.73 to 5.05	−9.77 to 6.20	−8.73 to 8.10	−9.60 to 7.58	0.36 to 1.07	0.41 to 1.25	0.38 to 1.25
Fourth	1.82	2.61	1.70	−1.81	−0.40	−2.71	0.89	1.03	0.94
	−3.99 to 7.62	−3.22 to 8.44	−3.88 to 7.29	−11.49 to 7.88	−10.58 to 9.79	−12.41 to 6.99	0.36 to 1.42	0.40 to 1.67	0.33 to 1.54
Most deprived	6.19	5.79	3.16	7.28	7.43	2.83	1.10	1.17	0.97
	−2.74 to 15.13	−0.3.47 to 15.05	−5.64 to 11.97	−4.41 to 18.98	−4.58 to 19.45	−8.68 to 14.33	0.28 to 1.91	0.26 to 2.08	0.12 to 1.83
Northern Ireland									
Least deprived (reference category)									
Second	−4.56	−4.16	−4.15	−5.30	−4.89	−4.46	0.89	0.93	1.00
	−12.60 to 3.48	−12.14 to 3.82	−11.98 to 3.69	−17.79 to 7.19	−17.09 to 7.30	−16.49 to 7.57	0.20 to 1.58	0.21 to 1.66	0.23 to 1.77
Third	−2.06	−2.09	−2.77	0.12	−0.33	−1.46	1.35	1.28	1.16
	−10.06 to 5.94	−9.94 to 5.77	−10.37 to 4.84	−12.94 to 13.17	−12.76 to 12.10	−13.93 to 11.00	0.32 to 2.38	0.29 to 2.26	0.24 to 2.09
Fourth	−0.48	−0.42	−2.28	−0.16	−0.18	−1.60	0.99	0.96	0.82
	−7.31 to 6.35	−6.97 to 6.14	−8.71 to 4.14	−11.66 to 11.34	−10.91 to 10.55	−12.07 to 8.86	0.24 to 1.73	0.25 to 1.66	0.25 to 1.38
Most deprived	−0.64	−0.11	−3.37	2.17	2.95	−2.52	1.11	1.17	0.86
	−7.68 to 6.40	−6.87 to 6.64	−9.95 to 3.21	−10.00 to 14.33	−8.58 to 14.48	−13.35 to 8.30	0.42 to 1.79	0.49 to 1.84	0.35 to 1.38
*Intraclass correlation (ICC) (%)*	3.86	3.68	3.31	3.75	3.74	3.19	4.84	5.27	5.36

For counts per minute and moderate to vigorous physical activity, percentage changes between geometric means of activity associated with varying levels of the covariates of interest are presented. For activity adherence, ORs (95% CI) for children, meeting the recommended guidelines are presented.

Model 1 has been adjusted for gender and season.

Model 2 has been additionally adjusted for confounders (child's ethnic group and child's BMI).

Model 3 has been adjusted for gender, season, child's ethnic group, child's BMI, and the following if significant in models 1 and 2: TV in child's bedroom, access to a garden, car usage, lone motherhood, home ownership, maternal socioeconomic circumstances, maternal education, Organisation for Economic Co-operation and Development (OECD) below 60% median poverty indicator, good area to bring up children, urban/rural and Index of Multiple Deprivation.

Significance levels: * <0.05, ** <0.01, *** <0.001.

“−”: Negative sign indicate decrease in physical activity.

BMI, body mass index; TV, television.

Measures of the home environment indicating socioeconomic disadvantage (lone motherhood, non-ownership, lower levels of maternal occupation, education and income) were associated with higher levels of physical activity, which persisted after adjusting for child's ethnic group and BMI. However, these associations were attenuated to non-significance in fully adjusted models, except for the association with maternal education (table 2).

### Neighbourhood environmental measures

Perceiving the neighbourhood to be a poor or very poor place to raise children was associated with more physical activity in children (total and MVPA only) but this was not significant in the fully adjusted models. No other variables describing the physical environment of the neighbourhood (access to play areas, perceptions of safety and whether urban or rural) were associated with physical activity.

Children's physical activity increased with increasing level of deprivation as indicated by the country-specific IMD quintile for England only. This association was not significant in the fully adjusted models for all outcomes. The IMD for Wales, Scotland and Northern Ireland was not significantly associated with children's physical activity.

At the individual-level of analysis, the overall performance of the models in terms of the percentage of the variation of the dependent variables explained by the variation of the predictor variables was approximately 12% in the unadjusted models and 14% in the final adjusted model for total activity. Equivalent figures for MVPA were 13% and 16%. In all models, the ICCs indicated that statistically significant proportions of the variation in physical activity were explained by variation at the area level for all models. For example, in total activity, 3.31% was explained by IMD when adjusting for gender and season and environmental characteristics that were significant in models 1 and 2.

## Discussion

### Statement of principal findings

In this large population-based study, accelerometer-measured physical activity in 7-year-old children was significantly associated in unadjusted analyses with characteristics of the physical and socioeconomic environments, for the home and neighbourhood. For the home environment, we found that children living in a family with no cars, those living in relatively disadvantaged circumstances had higher levels of physical activity. At the area level, more physical activity was associated with higher deprivation (IMD) for England only and with parental perceptions of it being a poor area for children. There was no association with rurality. In general, relationships were independent of child's BMI and ethnic group and were more likely for total activity and MVPA than for adherence to guidelines. However, when indicators of the environment were considered together, the only factors that remained significant were no cars in the household, lower levels of maternal education and a TV in the child's bedroom (for counts/min only), all associated with increased physical activity. This suggests that the dominant effect of the environment on physical activity is through socioeconomic characteristics related to personal assets (having the use of a car or higher maternal education). That having a TV in the child's bedroom should be associated with higher level of physical activity is counter-intuitive. However, in this sample having a TV in the child's bedroom was more common in less advantaged families, and so it may be acting as a marker of disadvantage rather than being on a causal pathway through sedentary behaviour. We also tested the hypothesis of whether having a TV in the child's bedroom would reduce wear time (eg, evening) in a way that could raise the average activity per observed minute and found no effect on wear time and therefore physical activity levels. For all associations, effect sizes associated with activity appeared to be modest but these are difficult to compare with other studies due to differences in methods of analysis, measures and sample characteristics.^16^

### Strengths and limitations

Strengths of this study include its large national and representative sample and the use of accelerometers to provide more objective measures of physical activity. To the best our knowledge this is the first study to investigate the influence of the physical and socioeconomic environments measured at the home and area level on children's physical activity. For some variables, such as access to a garden, there was little variability to analyse. Furthermore, although the range of measures was broad, many may be limited in their capacity to describe the environment and may, at the same time, be characterising personal traits of those who live in environments, rather than or as well as, the environment itself. This challenge is inherent to this field of study and we attempted to address it through our analytic strategy.

Acknowledged limitations include inability of accelerometers to measure certain types of activities, including aquatic activities, cycling and activities mainly requiring upper-body movement as well as to capture contexts of physical activity (eg, walking while carrying a load or walking uphill). In addition, self-reported measures of the physical and socioeconomic environments may result in bias, although patterns of associations with physical activity were reasonably consistent, at least in direction of effect, across different measures of the environment.

### Comparison with other studies

Our study supports earlier evidence reporting environmental influences on children's physical activity. However, while few other studies have examined reported and objective measures of the physical and socioeconomic environments^6^ ^10^ ^11^ only, Roemmich *et al*^10^ and McCormack *et al*^11^ adjusted for socioeconomic aspects of the environment. Current research has also explored the association between physical activity and the socioeconomic environment among adolescents;^7^ ^11^ however, the relationship among children is less clear.^34^ To the best of our knowledge, this is the first study of school-age children that examines individual and neighbourhood characteristics of the physical and socioeconomic environments. For most health behaviours, socioeconomic advantage is associated with health enhancing behaviours.^35^ For physical activity among children we found the reverse. Other evidence on this is mixed.^36^

There has been a recent focus on the independent contribution of sedentary behaviour on children's health^37^ and TV viewing has been used as a proxy for sedentary behaviour.^10^ ^12^ However, we found that children with a TV in their bedroom were more physically active. Only a few studies have investigated the association between number of TV sets at home and sedentary behaviour^10^ ^38^ ^39^ and from those, only Roemmich *et al*^10^ found a positive association. However, no study has reported the association between TVs in the home and physical activity. Our findings may indicate that sedentary and physically active lifestyles coexist (the ‘Active Couch Potato’^40^). Alternatively, having a TV in the child's bedroom could be a proxy of socioeconomic disadvantage^41^ or some other pathway, which is particularly associated with physical activity. Children's health behaviours develop first within the family environment and factors such as access to media may be important influences on children's sedentary and active behaviours.^41^

Access to a car could be another indicator of affluence as well as a disincentive to active travel. Our finding, that children who lived in households that used one or more cars were less active compared with those in households with no cars, agrees with current literature indicating that not having a car is an indicator of lower socioeconomic status and walking as a mode of transport.^42^

Maternal perceptions of neighbourhood safety were not associated with children's activity. This is consistent with some previous studies.^43^

Other studies have reported significant associations between reported or objective measures of the neighbourhood and physical activity,^4^ ^5^ ^11^ mainly focusing on the physical environment, of which we had few measures. We were able to examine neighbourhood social deprivation and found that it predicted higher levels of physical activity although not after accounting for individual characteristics. Neighbourhood deprivation is likely to be associated with families having lower levels of assets. Only a recent North American study has examined the association between neighbourhood deprivation and children's physical activity and findings are mixed.^44^ They found a strong association between higher neighbourhood deprivation and lower physical activity among African-Americans, but less consistent associations in white adolescents.

### Implications for research, policy and practice

Better measures are needed of the environment, for the home and the neighbourhood and to describe aspects related to physical and socioeconomic influences. Such measures need to discriminate between predictors of physical activity that relate to places (homes and neighbourhoods) and those that relate to people who live in those places. Analysis should also consider how people and the places where they live interact to affect health-enhancing behaviours. This study may provide a starting point, but methodological development is needed to determine causal pathways and potential interventions.

Increasing activity levels in children is a public health priority.^45^ The results of our study show that, although both physical and socioeconomic environments are associated with children’s physical activity, much of the variation appears to be determined close to home rather than in the wider environment.

**What is already known on this subject?**

- There is conﬂicting evidence on the association between the physical and socioeconomic home and neighbourhood environments and physical activity in children.
- These conflicts may be due to limitations in study design or the environmental measures used as most studies are small, have focused on the association between physical activity and the physical environment and have used self-reported measures of physical activity.

**What this study adds?**

- This is the first study to explore the influence of characteristics of the home and neighbourhood environments on accelerometer-measured physical activity in children, taking account of family socioeconomic circumstances and using measures that reflect physical and social characteristics of the neighbourhood.
- Higher levels of children's physical activity were associated with measures indicating disadvantage, at family and neighbourhood level. When adjusted for physical and socioeconomic correlates, the factors remaining significant were: household car usage and maternal education.
- The results of our study suggest that the dominant effect of the environment on physical activity is through home socioeconomic characteristics rather than the wider environment.
